# Investigating the Impact of Flap Overdesign on Viability

**DOI:** 10.1055/s-0036-1584263

**Published:** 2016-06-06

**Authors:** Andrew S. Aherrera, David J. Pincus, Adam J. Vernadakis

**Affiliations:** 1 Department of Plastic and Reconstructive Surgery, Lahey Hospital and Medical Center, Burlington, Massachusetts

**Keywords:** flap, overdesign, flap necrosis, viability, reconstructive surgery, tissue defect

## Abstract

**Background** Partial or complete flap necrosis is a detrimental outcome complicating reconstructive surgery. The purpose of this study was to evaluate the impact of flap overdesign on viability in the rat model.

**Methods** Forty Sprague-Dawley rats were equally divided into four groups receiving flaps of varying length-to-width ratios: 2:1, 3:1, 4:1, and 5:1. All animals had caudally based, modified McFarlane-style flap created. Areas of survival were assessed 14 days postoperatively and compared among groups using one-way analysis of variance.

**Results** The mean areas of flap survival were 8.0 ± 0.0 cm ^2^ , 7.8 ± 1.1 cm ^2^ , 8.3 ± 1.1 cm ^2^ , and 8.1 ± 1.5 cm ^2^ for the 2:1, 3:1, 4:1, and 5:1 length-to-width ratio groups, respectively. There were no statistically significant differences in mean areas of flap survival among groups ( *p* > 0.05).

**Conclusion** Flap overdesign does not increase the risk of flap necrosis in a random-pattern flap.

Partial or complete necrosis is a detrimental outcome complicating reconstructive surgery. Larger defects requiring coverage may be especially challenging and require a thorough understanding of skin flap anatomy and physiology, as well as careful flap design to achieve excellent functional and aesthetic outcomes. Many guidelines that govern flap design today have evolved from clinical experience, eventually solidified by practice and tradition. Length-to-width ratios, for instance, are one of the most established principles of plastic surgery and have long been used as a rough guide in flap design efforts despite a lack of experimental research paralleling the use of conventional arithmetic dictum.

Theoretically, flaps created under similar conditions of blood supply will survive to similar lengths regardless of width, but anecdotal evidence suggests that this result may not always be the case. 1 The inexplicable necrosis of large flaps with a wide base as well as small flaps with seemingly safe dimensions is an undesirable—albeit universal—experience among plastic surgeons. Furthermore, although the viability of overdesigned skin flaps remains incompletely characterized, it has nevertheless been clinically observed to result in otherwise avoidable necrosis along with significant consequences for patients.

To pursue this concern further, we sought to investigate the effect of increasing flap length while keeping a constant base width on overall skin flap survival, with the overall aim of elucidating the impact of flap overdesign on viability using the rat model. We hypothesized that overdesigned flaps, represented by greater length-to-width ratios, would result in proportionally diminished areas of flap survival.

## Materials and Methods

### Experimental Design

Forty Sprague-Dawley rats weighing an average of 303.3 ± 14.3 g (range 270 to 330 g) were used in this study, which was approved by the Institutional Review Board and conducted according to the animal care guidelines outlined in the National Institutes of Health Guide for the Care and Use of Laboratory Animals. 2 Rats were randomly divided into four groups of 10 and underwent creation of caudally based, modified McFarlane-style flaps of varying length-to-width ratios: 2:1, 3:1, 4:1, and 5:1. 3 4 All 40 rats were examined daily until they were euthanized 14 days following flap creation. The maximum amount of individual flap viability in each rat was determined, and the mean areas of flap survival were compared among the four groups.

### Surgical Procedure

**Fig. 1:**
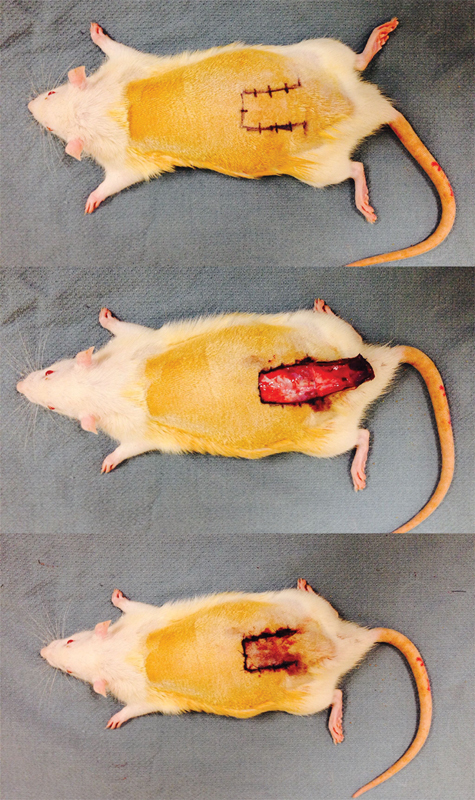
Preoperative markings for the dorsal, caudally based, random-pattern flap (top). The undersurface of the flap is seen with the blood supply arising from its base at the caudal portion of the flap (center). The flap has been replaced in its bed and sutured into place (bottom).

All procedures were performed under sterile conditions, with all rats adequately anesthetized using 120 mg/kg of intraperitoneal ketamine and 7.5 mg/kg of intraperitoneal xylazine. The anatomical boundaries of the modified McFarlane-style flaps were defined inferiorly by the posterior superior iliac spines and were centered using the spinal column as a point of reference ( Fig. 1 ). Each flap was designed based on a width of 2 cm, and as such, the 2:1, 3:1, 4:1, and 5:1 length-to-width ratio groups had flap lengths of 4, 6, 8, and 10 cm and total areas of 8, 12, 16, and 20 cm ^2^ , respectively.

Incisions were made along the superior and lateral borders, and each flap was completely elevated to its base along the caudal border. Blunt and sharp dissection was used to raise the panniculus carnosus from the underlying deep fascia, and hemostasis was ensured using bipolar cautery as needed. Undermining immediately distal to the caudal border of at least 1 cm was performed to ensure that there were no intact perforating vessels that may have directly supplied each flap. The flaps were then replaced in their beds and sutured in place with interrupted 4–0 nylon sutures.

### Calculating Mean Areas of Flap Survival

**Fig. 2:**
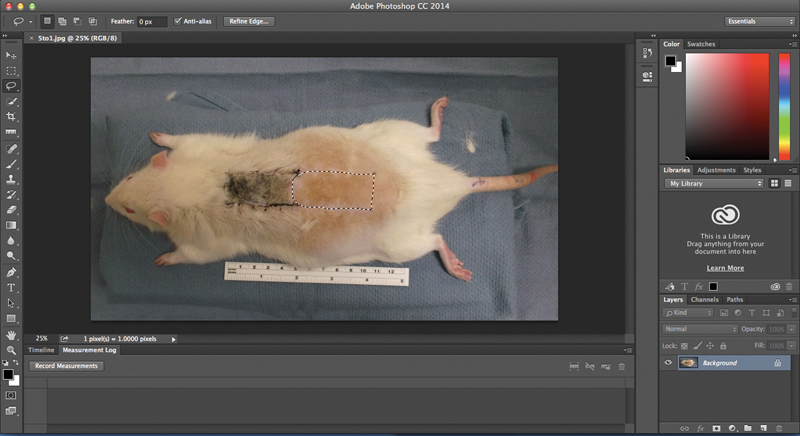
Adobe Photoshop (Adobe Systems, Inc., Mountain View, California, United States) was used to analyze the digital photographs to determine the area of flap survival in each animal. The lasso tool was used to outline the viable portion of the flap to yield the pixelated area of this irregularly shaped region.

Prior to euthanization, all rats were adequately anesthetized using 120 mg/kg of intraperitoneal ketamine and 7.5 mg/kg of intraperitoneal xylazine for the flaps to be properly evaluated. The outer boundaries of each flap were outlined, as were the necrotic portions, identified according to appearance, pliability, and texture indicative of nonviability as described by prior investigators. 4 5 Digital photographs were taken of each flap, and all images were analyzed using Adobe Photoshop version 2014.2.1 (Adobe Systems, Inc., Mountain View, California, United States). With the use of this program, pixelated areas of the viable regions of interest were obtained to determine the area of survival for each flap ( Fig. 2 ). Photographs were evaluated as a team by all investigators whereby necrotic and nonnecrotic areas were delineated based on consensus.

### Statistical Analysis

The mean areas of flap survival were compared between groups using one-way analysis of variance. The Levene test was used to confirm the equality of variances, and the Tukey method was used for post hoc comparisons. Analyses were performed using SPSS version 22 (IBM Corporation, Armonk, New York, United States). The mean areas of flap survival are given for each experimental group along with standard deviations.

## Results

**Fig. 3:**
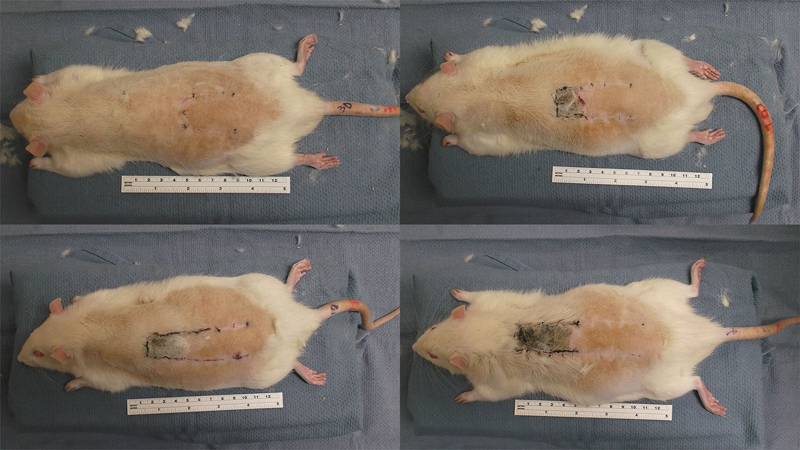
Dorsal, caudally based, random-pattern flaps of one rat from each experimental group are shown: 2:1 group (top, left); 3:1 group (top, right); 4:1 group (bottom, left); 5:1 group (bottom, right).

**Fig. 4:**
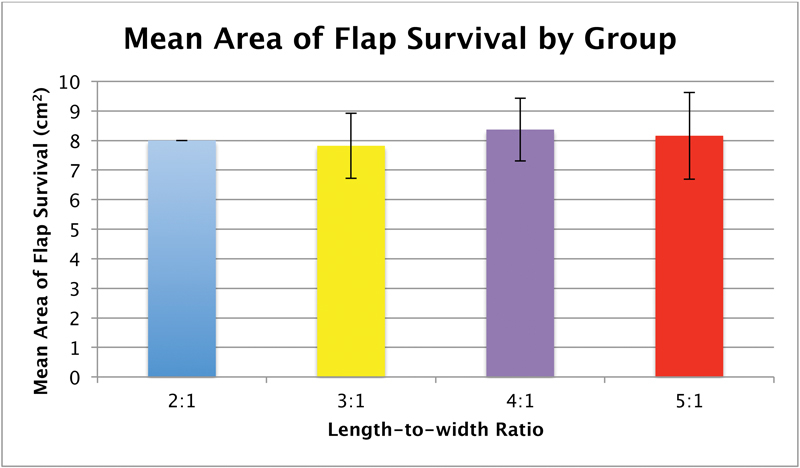
The mean area of flap survival and standard deviations are shown for each group.

Each experimental group demonstrated nearly consistent flap survival beginning at the caudalmost portion and extending a consistent measure along the distal length of the flap ( Fig. 3 ). The mean areas of flap survival were 8.0 ± 0.0 cm ^2^ , 7.8 ± 1.1 cm ^2^ , 8.3 ± 1.1 cm ^2^ , and 8.1 ± 1.5 cm ^2^ for the 2:1, 3:1, 4:1, and 5:1 length-to-width ratio groups, respectively ( Fig. 4 ). Statistical analysis revealed no significant differences in the mean areas of flap survival among all four groups ( *p* > 0.05).

## Discussion

Skin flap necrosis is an undesirable outcome that has significant consequences for patients. Even the most experienced plastic surgeons have encountered this complication despite meticulous utilization of design fundamentals. Nevertheless, an awareness of the various factors that influence flap viability is necessary to improve the odds of successful reconstructive efforts. This investigation was conducted in an attempt to offer some experimental evidence regarding the impact of flap overdesign on tissue viability. We initially hypothesized that overdesigned flaps would result in proportionally diminished areas of flap survival, but based on our findings, it appears that flap overdesign does not impact the viability of random-pattern flaps in the rat model.

In theory, the survivability of the distal portion of a flap depends on the physical properties of its supplying vessels and their perfusion pressures. 6 Random-pattern flaps derive their vascular supply from the subdermal plexus rather than a named skin perforator. When the perfusion pressure along the length of the flap falls below the critical closing pressure of the arterioles in the subdermal plexus, blood flow ceases and tissue ischemia ensues. 7 Although now considered to be a fallacy, the viable length of a flap was originally believed to be entirely dependent on the width of its base—by widening the base, more supplying vessels could be incorporated into the flap and support a greater surviving length. However, the intravascular resistance of the additional subdermal arterioles remains the same, and flap ischemia will occur at the same location along the length of the flap regardless of the total number of supplying vessels incorporated. 1 As such, perfusion pressure, rather than the classic concept of length-to-width ratios, dictates flap survival.

The metabolic demands of a flap also contribute to ischemia and tissue necrosis. Experimental research has demonstrated that glucose levels decrease and lactate levels increase distally along the length of a flap, which may be reflective of decreased supply of nutrient-rich blood or increased utilization by tissue further away from the base of the flap. 8 9 Postoperatively, adenosine triphosphate levels rapidly fall throughout the entire flap but more so in the most ischemic distal regions. Additionally, the levels of cyclic adenosine monophosphate, which is derived from adenosine triphosphate and serves as a marker for increased cellular metabolism, begin to markedly increase throughout the flap at around 12 hours postoperatively. 10 The width of the flap may be equated to its vascular supply and the entire area of the flap to its vascular demand. A stable equilibrium between supply and demand would therefore be disrupted by changes in length while maintaining a constant width.

In practice, length-to-width ratios are still used as rough guidelines in flap design, which vary by anatomic site. In the lower extremity, optimal length-to-width ratios are not to exceed 2:1. In the head and neck area, 3:1 and even 4:1 length-to-width ratios may be accommodated by its richer vascularity. Intuitively, the regions of flaps exceeding the accepted dimensions dictated by the appropriate length-to-width ratio are less likely to be viable. Moreover, it would seem logical that flaps designed over and beyond these accepted dimensions would result in even greater areas of necrosis that diminish the expected areas of viability in accordance with the increased metabolic demand. Interestingly, however, the results of our study do not reflect this concept—overdesigned flaps did not contribute additional necrosis sufficient to result in diminished areas of viability in comparison with flaps of smaller dimensions. Still, it should be noted that our results demonstrate greater variability (as evidenced by greater standard deviations) in the mean areas of flap survival among the 3:1, 4:1, and 5:1 length-to-width ratio groups, and the 2:1 length-to-width ratio group exhibited the most consistent flap survival.

The findings presented in this study support one of two possibilities. Given that accepted length-to-width ratios in humans vary by anatomic site, it is possible that the threshold for overdesign using dorsal, caudally based, random-pattern flaps in the rat model exceeds a length-to-width ratio of 5:1. Alternatively, perhaps our findings validate the triumph of physiology over arithmetic; given that flap survival length is dependent on the physical properties of its supplying vessels and their perfusion pressures, it is possible that flaps created under similar conditions of vascular supply will survive to similar lengths irrespective of their overall dimensions. Regardless, the findings presented herein challenge conventional dogma and question the rigidness of established length-to-width ratios. Furthermore, these results represent a novel stride toward characterizing the viability of overdesigned skin flaps and lend support to pushing the boundaries of established practice patterns as they pertain to flap overdesign in reconstructive surgery.

Our study has several shortcomings and, like most studies that are among the first to experimentally explore a complex clinical quandary, perhaps raises as many questions as it answers. Primarily, and as with all animal studies, it is impossible to say how closely rat models equate with humans. Still, the numerous prior investigations on skin flap physiology have established the utility and translational relevancy of rat models in better understanding cutaneous ischemia, wound healing, and skin flap metabolism in humans. 3 4 5 9 Given that a rigidly controlled, prospective, randomized trial of flap design in humans would be challenging, animal studies will have to suffice. Furthermore, although current practice patterns in evaluating flap viability predominantly rely on subjective visual and tactile methods, future investigations utilizing microangiographic, scintigraphic, or histologic evaluations would also be beneficial in further elucidating the impact of flap overdesign on viability.

## Conclusion

Despite its inherent limitations, that data presented in this article represents the first experimental evidence regarding the impact of flap overdesign on the viability of random-pattern skin flaps. We conclude that flap overdesign does not increase the risk of additional flap necrosis in a random-pattern flap and that the length-to-width principle may not be as rigid and uncompromising as conventionally thought. This information should aid plastic surgeons in flap design efforts, but additional research is essential before more specific guidelines may be defined to further solidify practice patterns that ameliorate the challenging dilemma of partial or complete necrosis of skin flaps in reconstructive surgery.
